# Autobiographical Memory and Episodic Specificity Across Different Affective States in Bipolar Disorder

**DOI:** 10.3389/fpsyt.2021.641221

**Published:** 2021-05-07

**Authors:** Rafael de Assis da Silva, Marcelo Baggi Tancini, Renata Lage, Rodrigo L. Nascimento, Cristina M. T. Santana, J. Landeira-Fernandez, Antonio Egidio Nardi, Elie Cheniaux, Daniel C. Mograbi

**Affiliations:** ^1^Department of Psychology, Pontifícia Universidade Católica-Rio, Rio de Janeiro, Brazil; ^2^School of Medicine and Surgery, Federal University of the State of Rio de Janeiro (Unirio), Rio de Janeiro, Brazil; ^3^Institute of Psychiatry, Federal University of Rio de Janeiro, Rio de Janeiro, Brazil; ^4^Faculdade de Ciências Médicas, State University of Rio de Janeiro (UERJ), Rio de Janeiro, Brazil; ^5^Institute of Psychiatry, Psychology and Neuroscience, King's College London, London, United Kingdom

**Keywords:** bipolar disorder, autobiographical memory, episodic memory, Mood dependent memory, Mood congruent memory

## Abstract

Autobiographical memory is essential to ground a sense of self-identity, contributing to social functioning and the development of future plans, and being an essential source for the psychiatric interview. Previous studies have suggested loss of autobiographical episodic specificity in unipolar depression, but relatively fewer investigations have been conducted in bipolar disorder (BD) patients, particularly across different mood states. Similarly, there is a scarcity of systematic investigations about mood-congruent and mood-dependent memory in relation to autobiographical memory in BD. Considering this, a total of 74 patients with BD (24 in euthymia, 26 in mania, and 24 in depression) responded with autobiographical memories to cue words belonging to four categories: mania, depression, BD, and neutral. Episodic specificity was scored according to the Autobiographical Interview, with high intra- and inter-rater reliability. Results indicated that patients in mania generally re-experience more episodic details than those in depression. Depressed bipolar patients reported fewer details of perception and less time integration of memories than those in euthymia or mania. Words linked to depression and BD induced greater episodic re-experiencing than neutral words, just as words about BD provided greater episodic re-experiencing and more details of emotion/thoughts than words about mania. Words linked to depression provoked more time details about the recalled episodes than words on BD or neutral themes. No mood-congruent or mood-dependent effects were observed. Current findings may improve the ability of clinicians to conduct psychiatric interviews and the diagnosis of BD, with special attention to how memory details are generated across different mood states of the condition. Additionally, interventions to foster autobiographical recollection in BD may be developed, similar to what has already been done in the context of schizophrenia.

## Introduction

Autobiographical memory can be defined as the ability to recall memories that are personally important and often emotionally charged (1). It serves a variety of important purposes, such as optimizing social functioning, developing future plans based on the past, and maintaining an individual conception of identity (2). Impairments in autobiographical memory are typically associated with difficulties in executive functions, such as metacognition, monitoring, and inhibitory control (3). In addition, given that autobiographical memory represents the main source for patients to describe their past experience, its impairments may have an important impact on the clinical interview and diagnosis of psychiatric conditions.

Patients with major depressive disorder suffer from impaired ability to recall autobiographical memories, with difficulties in remembering specific events—the phenomenon of memory generalization (4), which can be observed clinically in patients reporting difficulties and sad moments without being able to provide specific details to anchor their vague memories. Similar impairments have also been found in patients diagnosed with schizophrenia (5–7). For example, these patients show a lower ability to recall specific episodes of autobiographical memory when compared to the general population. Additionally, it has been shown that major affective disorder may have an impact on sensory processing (8).

Autobiographical memory has been comparatively less studied in bipolar disorder (BD). BD affects ~30 million people worldwide and is characterized by severe mood swings that include periods of elevation (mania) and depression, intertwined with periods of remission (euthymia) (9, 10). Patients with BD are 12 × more likely to commit suicide than healthy individuals (11), which may be linked to childhood maltreatment (12). The literature suggests that patients diagnosed with BD have impaired autobiographical memory even when in the euthymic phase (13). These patients experience impairment in the process of encoding memories when in euthymia, with consequent difficulty recalling them in a later moment (13). However, few studies have explored the level of episodic richness in the memories of patients with BD, in particular across different mood states.

The effect of mood in memory retrieval has also been explored in relation to mood-congruent and mood-dependent memory (14). Mood-congruent memory is the phenomenon of individuals tending to recall memories with the same affective valence as the one he/she is experiencing in the present (for example, a depressed individual tending to remember negative facts of his past). By contrast, mood-dependent memory refers to an individual's ability to assimilate neutral memories in a given affective state and the tendency to recall them when in a state similar to the original one (e.g., remembering better memories that occurred during mania when in mania again). Few articles, however, have tested the relationship between the affective state in which memories were stored and the ability of BD patients to recall them. For instance, there has been some evidence that BD patients have impaired ability to recall events which were stored during the manic phase (15). Nevertheless, a systematic investigation of the topic has not yet been published.

Considering the relevance of autobiographical memory for the assessment and diagnosis of psychiatric conditions, its role in the formation of a sense of self and identity, and the relative scarcity of the literature on this topic, the current article investigates the relationship between autobiographical and BD. Specifically, this study explores the ability of patients diagnosed with BD to recall autobiographical details across different mood states. In addition, the effects of mood-congruent and mood-dependent recollection on autobiographical memory of BD patients were also investigated, using cue words belonging to specific categories that referred to BD, mania and depression. It is hypothesized that bipolar depression will lead to impoverished episodic recall, similar to unipolar depression. Additionally, it is predicted that mood-congruent and mood-dependent effect will be observed (e.g., patients in mania remembering more vividly episodes linked to mania cue words).

## Methods

### Sample

This study was performed in an outpatient research clinic in the Institute of Psychiatry of the Federal University of Rio de Janeiro. Inclusion criteria were: diagnosis of BD type I or type II; age over 18 years; written informed consent. Exclusion criteria were refusal to participate in the research, non-cooperation during the application of assessment instruments and presence of severe non-psychiatric Illness. A total of 74 patients were included in the study (24 in euthymia, 26 in mania, and 24 in depression). The local research ethics committee approved the study (ref #4223214).

### Clinical Evaluation

The psychiatric diagnosis in this study was formulated according to DSM-5 criteria (16) and through clinical evaluation conducted by a psychiatrist. Sociodemographic data (age, sex, and educational level) were collected. The affective state of patients was determined through DSM-5 criteria, with the following scales being used:

- Hamilton Depression Scale [HAM-D; (17)]—The HAM-D includes 17 items assessing the severity of depression, with scoring on items ranging from 0 to 4 or 0 to 2. Scoring is based on patient self-report and clinician observation. Higher HAM-D scores indicate more severe depressive symptoms.
- Young Mania Rating Scale [YMRS; (18)]—The YMRS comprises 11 items to assess manic symptoms. Scoring on items range from 0 to 4, with four items being graded from 0 to 8. Scoring is based on patient self-report and clinician observation. Higher YMRS scores indicate more severe manic symptoms.
- Clinical Global Impressions Scale for bipolar illness (19)—CGI-BP presents global scoring pertaining to the severity of the affective episode. Based on clinical assessment, severity is scored from 1 (normal) to 7 (very severely ill).

In addition, the presence of psychotic symptoms was ascertained when at least one delusional narrative or hallucination of any kind was detected.

### Autobiographical Memory

The patients were asked to remember the first autobiographical memory that came to mind after the interviewer said a cue word. The task used 12 words, divided into four three-word categories: mania (“aggressive,” “fast-talking,” “agitation”); depression (“demotivated,” “tired,” “depressed”); bipolar disease (“hospital,” “medication,” “hospitalization”); and neutral (“look,” “afternoon,” “stroll”). After the initial description of the memory, participants were asked when and where each memory happened. The interviews were recorded and fully transcribed for analysis.

The scoring method used was based on the Autobiographical Interview, by Levine and collaborators (20). Memories were classified according to the following details: time, place, perceptual, time integration, thought/emotion, and episodic richness, with each of these dimensions rated from 0 to 3, with the exception of episodic richness—rated from 0 to 6, to provide a finer grained scoring (20). That generated, for each cue word, total scores ranging from 0 to 21, and scores ranging from 0 to 3 in each dimension (or 0 to 6 in the case of episodic richness). Scores were summed across words belonging to the same category, yielding overall scores for mania, depression, bipolar, and neutral words.

Intra-rater (test-retest) reliability was established by rating twice 10% of the material with a gap of 2 months between ratings. Average agreement was 75.5% (range: 57.3-87.5%) with an average Cohen's kappa coefficient of 0.63 (range: 0.45-0.78; *p* < 0.001) indicating substantial agreement. Inter-rater reliability was assessed by a coder blind to group membership rating 10% of the material. Average agreement was 74.8% (range: 67.7-87.5%) with an average Cohen's kappa coefficient of 0.61 (range: 0.55-0.78; *p* < 0.001) indicating substantial agreement.

### Procedures

The study was conducted in a private room at the Bipolar Disorder Research Outpatient Unit of IPUB-UFRJ. After the initial clinical evaluation, patients completed the autobiographical memory interview.

### Statistical Analyses

Descriptive statistics were generated to illustrate sample characteristics. Patients in different affective states were compared in relation to sociodemographic and clinical variables with one-way ANOVAs (in the case of age, YMRS, HAM-D, and CGI-BP), followed by *post-hoc* pairwise comparisons, or Chi-square tests (in the case of gender, educational level, and presence of psychotic symptoms). Differences in autobiographical memory were explored with 3 x 4 mixed-design ANOVAs, with mood state as a between-subjects factor (euthymia, mania, or depression) and word category as a within-subjects factor (neutral, bipolar, mania, and depression). The ANOVAs were calculated for total memory detail scores, as well as for the subscores of episodic re-experiencing, time, place, perception, thought and emotion, and time integration, with effect sizes being reported as $\eta_{\text{p}}^{2}$ scores. Pairwise comparisons followed significant ANOVA effects. All analyses were run in SPSS v.24, with α = 0.05.

## Results

### Sample Characteristics

Sample characteristics can be seen in Table 1. There were no significant differences between groups for age [*F*_(2, 73)_ = 2.12, *p* = 0.128] and educational level [$\chi_{\left(2\right)}^{2}$ = 1.62, *p* = 0.446], but a significant gender difference [$\chi_{\left(2\right)}^{2}$ = 7.83, *p* = 0.020], with more women in the mania group. Regarding clinical variables, significant differences were found for mania [*F*_(2, 73)_ = 134.73, *p* < 0.001] and depression symptoms [*F*_(2, 73)_ = 133.45, *p* < 0.001], as well as for severity of illness [*F*_(2, 73)_ = 66.27, *p* < 0.001]. *Post-hoc* tests indicated higher YMRS scores in the mania group in relation to patients with depression and in euthymia (*p* < 0.001), with no differences between depression and euthymia (*p* = 0.584). Significantly higher HAM-D scores were found in the depression group in relation to both groups (*p* < 0.001), with patients in mania also showing more depressive symptomatology than patients in euthymia (*p* = 0.010). There were no significant differences in presence of psychotic symptoms between groups [$\chi_{\left(2\right)}^{2}$ = 5.07, *p* = 0.079]. Finally, both mania and depression groups had significantly higher illness severity than the euthymia group (*p* < 0.001), with no differences between them (*p* = 0.397).

**Table 1:** Socio-demographic and clinical characteristics of participants.

**Variable**	**Euthymia (*n* = 24)** **Mean (SD)/range**	**Mania (*n* = 26)** **Mean (SD)/range**	**Depression (*n* = 24)** **Mean (SD)/range**	**Group differences**
Age	49.9 (12.8)/33–79	53.6 (10.9)/41–75	46.4 (13.4)/23–70	–
Gender^*^	14/10	24/2	18/6	M > E = D
Educational level^**^	19/5	18/8	15/9	–
YMRS	1.5 (2.2)/0–7	20.7 (7.3)/12–40	2.2 (2.2)/0–7	M > E = D
HAM-D	0.9 (1.8)/0–7	3.6 (2.6)/0–10	17.1 (5.6)/8–28	D > M > E
Psychotic symptoms^***^	24/0	22/4	23/1	–
CGI global	1.2 (0.4)/1–2	3.9 (1.0)/3–6	4.1 (0.9)/3–6	E < M = D

* *#Female/Male;*

** *#Without/With higher education;*

*** *#Absent/Present; YMRS, Young Mania Rating Scale; HAM-D, Hamilton Depression Rating Scale; CGI, Clinical Global Impression scale*.

### Autobiographical Memory

**Figure 1:**
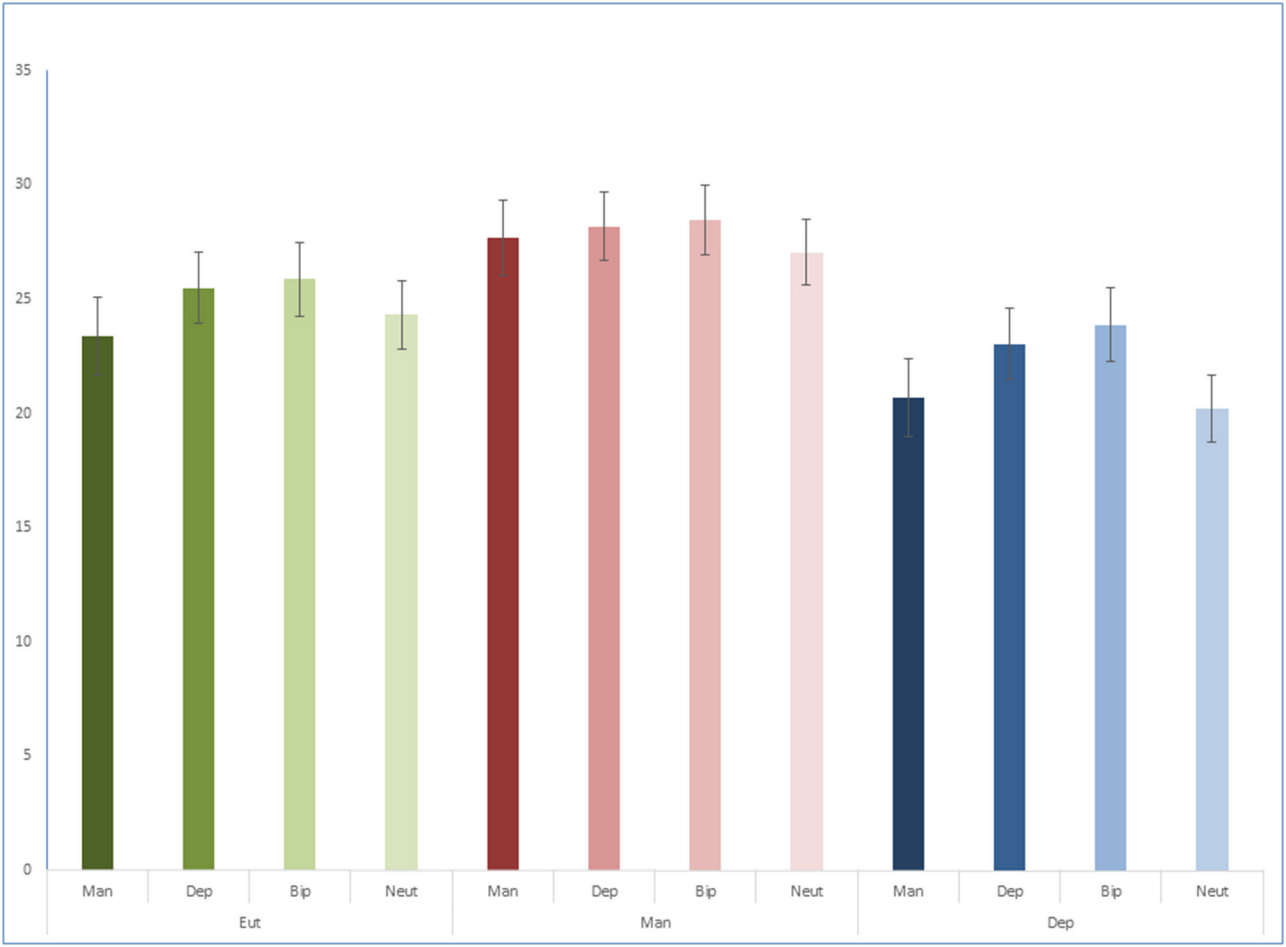
Total episodic detail scores.

For total scores (Figure 1) there was no significant interaction [*F*_(6, 213)_ = 0.46, *p* = 0.834, $\eta_{\text{p}}^{2}$ = 0.01], but significant main effects of mood state [*F*_(2, 71)_ = 5.32, *p* = 0.007, $\eta_{\text{p}}^{2}$ = 0.13] and word category [*F*_(3, 213)_ = 3.59, *p* = 0.015, $\eta_{\text{p}}^{2}$ = 0.05]. Pairwise comparisons indicated that patients in mania provided more episodic details than patients in depression (*p* = 0.002). Lower episodic detail was found for neutral words in relation to bipolar (*p* = 0.005) and depression words (*p* = 0.011), and for mania words in relation to bipolar words (*p* = 0.027).

**Figure 2:**
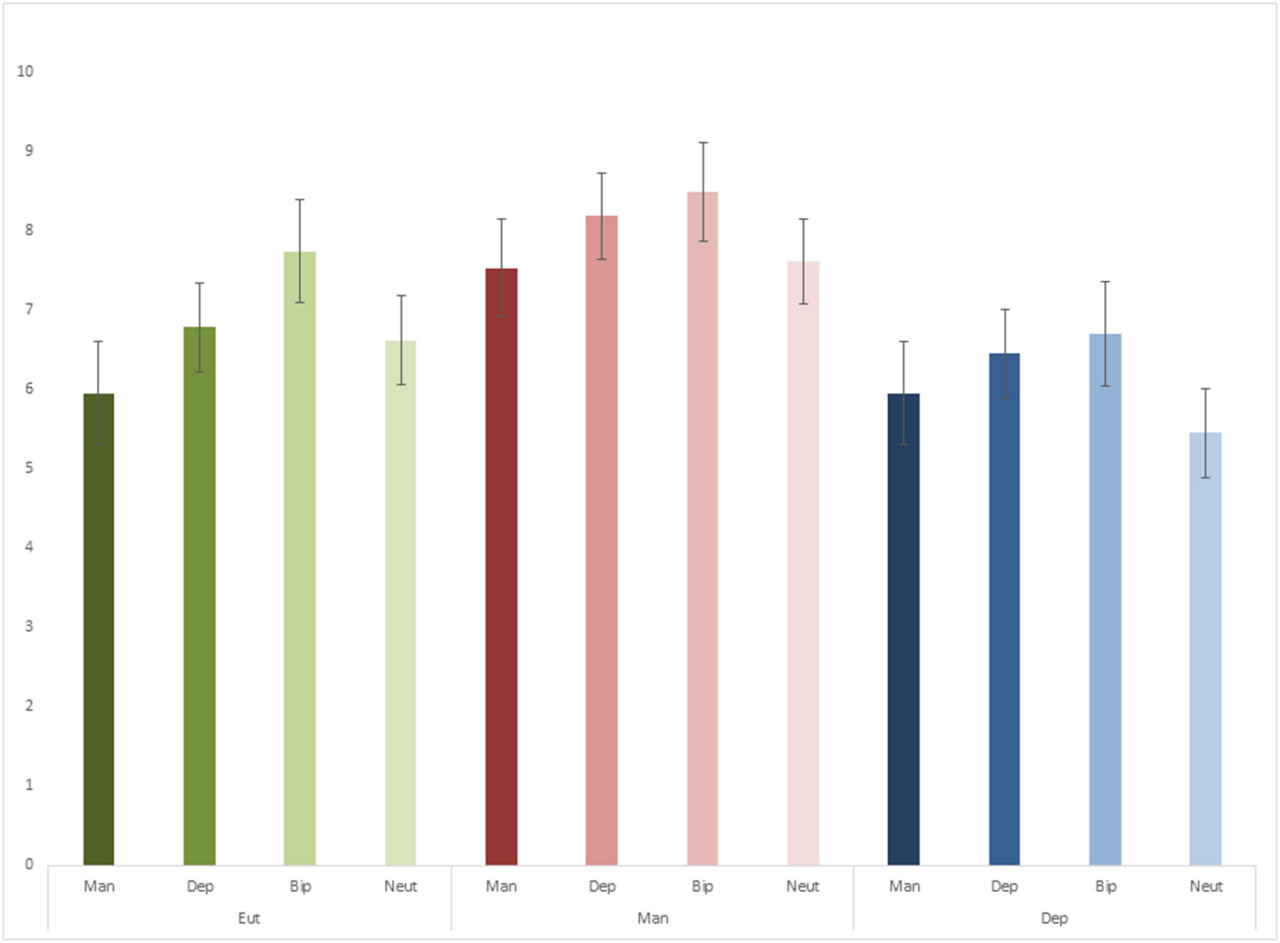
Episodic re-experiencing scores.

For episodic re-experiencing (Figure 2), there was no significant interaction [*F*_(6, 213)_ = 0.46, *p* = 0.835, $\eta_{\text{p}}^{2}$ = 0.01], but significant main effects of mood state [*F*_(2, 71)_ = 3.91, *p* = 0.024, $\eta_{\text{p}}^{2}$ = 0.10] and word category [*F*_(3, 213)_ = 4.92, *p* = 0.003, $\eta_{\text{p}}^{2}$ = 0.06]. Pairwise comparisons indicated that patients in mania re-experienced more episodic details than patients in depression (*p* = 0.008). Here again, lower episodic re-experiencing was found for neutral words in relation to bipolar (*p* = 0.002) and depression words (*p* = 0.025), and for mania words in relation to bipolar words (*p* = 0.004).

**Figure 3:**
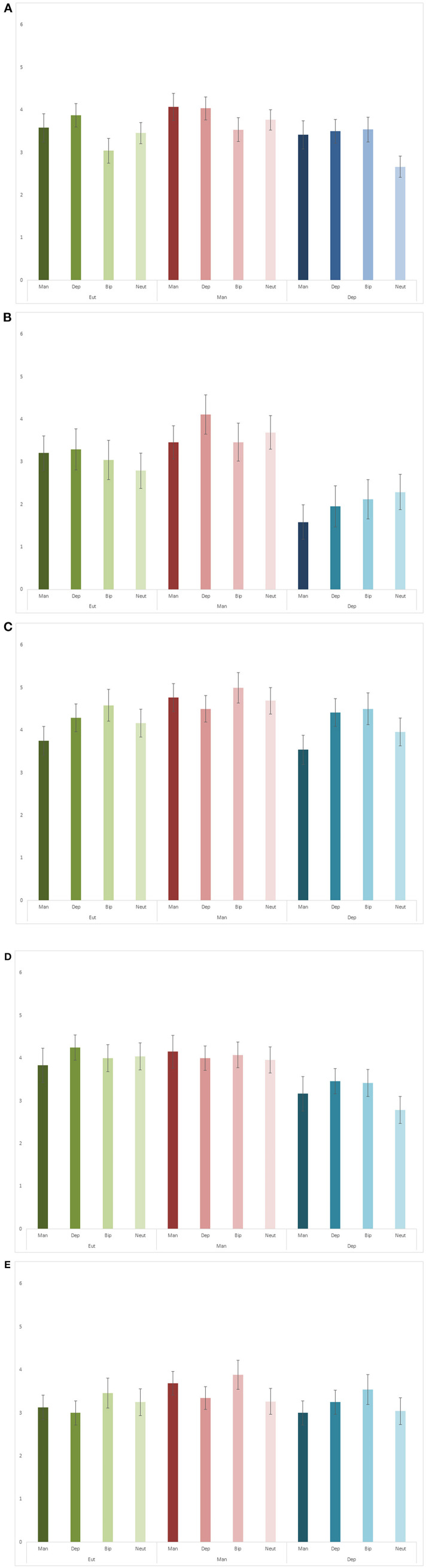
Time **(A)**, perceptual **(B)**, thought and emotion **(C)**, time integration **(D)** and place detail **(E)** scores.

For time details (Figure 3A), there was no significant interaction [*F*_(6, 213)_ = 1.78, *p* = 0.105, $\eta_{\text{p}}^{2}$ = 0.05] or main effect of mood state [*F*_(2, 71)_ = 1.94, *p* = 0.151, $\eta_{\text{p}}^{2}$ = 0.05], but a significant main effect of word category [*F*_(3, 213)_ = 3.72, *p* = 0.012, $\eta_{\text{p}}^{2}$ = 0.05]. Pairwise comparisons indicated that depression words resulted in more time details than bipolar (*p* = 0.016) and neutral words (*p* = 0.002).

For perceptual details (Figure 3B), there was no significant interaction [*F*_(6, 213)_ = 0.76, *p* = 0.599, $\eta_{\text{p}}^{2}$ = 0.02] or main effect of word category [*F*_(3, 213)_ = 0.67, *p* = 0.571, $\eta_{\text{p}}^{2}$ = 0.01], but a significant main effect of mood state [*F*_(2, 71)_ = 6.67, *p* = 0.002, $\eta_{\text{p}}^{2}$ = 0.16]. Pairwise comparisons indicated that patients in depression produced fewer perceptual details than patients in euthymia (*p* = 0.025) and mania (*p* = 0.001).

For thought and emotion details (Figure 3C) there was no significant interaction [*F*_(6, 213)_ = 0.88, *p* = 0.510, $\eta_{\text{p}}^{2}$ = 0.02] or main effect of mood state [*F*_(2, 71)_ = 2.04, *p* = 0.138, $\eta_{\text{p}}^{2}$ = 0.05], but a significant main effect of word category [*F*_(3, 213)_ = 3.29, *p* = 0.022, $\eta_{\text{p}}^{2}$ = 0.04]. Pairwise comparisons indicated bipolar words prompted more thought and emotion details than mania words (*p* = 0.012).

For time integration details (Figure 3D), there was no significant interaction [*F*_(6, 213)_ = 0.60, *p* = 0.732, $\eta_{\text{p}}^{2}$ = 0.02] or main effect of word category [*F*_(3, 213)_ = 0.83, *p* = 0.478, $\eta_{\text{p}}^{2}$ = 0.01], but a significant main effect of mood state [*F*_(2, 71)_ = 3.70, *p* = 0.030, $\eta_{\text{p}}^{2}$ = 0.09]. Pairwise comparisons indicated that patients in depression produced fewer time integration details than patients in euthymia (*p* = 0.024) and mania (*p* = 0.019).

There were no significant interactions [*F*_(6, 213)_ = 0.54, *p* = 0.780, $\eta_{\text{p}}^{2}$ = 0.01] or main effects [word category: *F*_(3, 213)_ = 2.56, *p* = 0.056, $\eta_{\text{p}}^{2}$ = 0.03; mood state: *F*_(2, 71)_ = 0.74, *p* = 0.481, $\eta_{\text{p}}^{2}$ = 0.02] for place details (Figure 3E).

## Discussion

The present study suggests that bipolar patients in mania generally re-experience more episodic details than those in depression. Depressed bipolar patients reported fewer details of perception and less time integration of memories than those in euthymia or mania. Words linked to depression and BD induced greater episodic re-experiencing than neutral words, just as words about BD provided greater episodic re-experiencing and more details of emotion/thoughts than words about mania. Words linked to depression provoked more time details about the recalled episodes than words on BD or neutral themes.

The literature has shown that the autobiographical recall of memory during depressive episodes is usually generalized to the detriment of more specific events (21). The result of the present study is in line with these findings, showing that depressed bipolar patients reported fewer details of episodes than those in mania. Individuals in mania may have hypermnesia, with an excess of memories occurring in a short period of time, though the same does not occur during depression.

The model described by Conway and Pleydell-Pearce (22) suggests that the search for specific information in depression is reduced after some generalized information is recovered. A possible cause for that is protecting the self from the threat of recovering negative information. This notion is in accordance with the concept of mnemonic interlock, described by Williams and Scott (23), which consists of a mechanism in which individuals may abort mnemonic research not reaching specific levels and stopping at a categorical level, with the aim of not getting in contact with painful memories.

Studies that evaluated autobiographical memory in other disorders, such as generalized anxiety disorder (24), obsessive-compulsive disorder (25), and borderline personality disorder (26), did not systematically detect a propensity to recover more generalized memories, unless there were comorbid depressive symptoms (27). Exceptions were noted only for post-traumatic stress disorder and acute stress disorder, in which overgeneralization was more consistently seen (28–30). A mechanism to avoid painful memories is possibly involved in both depressive states and disorders triggered by trauma.

However, traumatic experiences would not be the only predisposing factor for the overgeneralization cognitive style. A review conducted by Williams et al. (31) reported that, in addition to the traumatic experience, deficits in executive skills and depressive rumination could contribute to this low specificity. Williams and Scott (23) suggested that excessive memory encoding may influence dysfunctional recall during depressive episodes. Wheeler et al. (32) reported that reductions in working memory capacity, executive functioning in general, or of the supervisory attentional system, would be factors contributing to overgeneralization.

Mansell and Lam (33) also reported that individuals with BD in remission recall more generalized than specific memories for negative cue words when compared to a group of individuals with unipolar depression in remission and healthy controls. Our study also reports that groups of words with negative valence can influence details of the recalled episode. Words about depression provoked more time details than words on BD or neutral themes. It is possible that the words about depression lead to descriptions that extend over time (e.g., “for 3 months I was like that”; “every afternoon was the same”).

Our study suggests that depressed bipolar patients reported fewer perceptual details and less time integration of memories than those in euthymia or mania. The literature suggests that patients in depression may have hypoesthesia, with a global decrease in perceptual intensity (8). They would for example report a colorless world (34) or a decrease in the perception of food taste (35). It is also common for depressed patients in clinical settings to report time passing more slowly, with the time of their memories being fragmented and vague. A meta-analysis conducted by Thönes and Oberfeld (36) showed that depression has in fact moderate effects on the subjective time flow, while judgment on duration of events remains basically unchanged.

The words about BD yielded more details of emotion or thought than words on mania, regardless of the patient's affective state. This may refer to the high emotional value of some of the recalled episodes (e.g., hospital admissions). A study by King et al. (15) reported that, regardless of their affective status, BD patients were more likely to recall events from an observer's perspective than healthy controls, who preferred to rely on the field perspective. The authors suggest that patients with BD are possibly more likely to adopt the perspective of a detached observer when recalling past events, focusing less on emotions than on objective circumstances. Our study suggests that BD patients produce comparatively more emotionally charged memories, depending on the memory theme.

Regarding potential mood-congruent and mood-dependent effects, our results did not suggest any interactions between mood state and word categories (e.g., patients in mania remembering more vividly episodes linked to mania). There are two main hypotheses for these findings. It is possible that the impact of mood on autobiographical memory, such as the overgenerality observed during depression, overrides any congruency or dependence effects. Alternatively, the cue words may not have tapped into the correct constructs to generate mood-congruent and mood-dependent effects, but given the face validity of some of the terms chosen (e.g., “demotivated,” “tired,” “depressed” in the depression category), this is a less straightforward explanation.

This study has some limitations. The inclusion of a control group would have allowed the observation of possible general effects of BD in relation to autobiographical memory. For instance, the degree of impairment in euthymic patients in relation to healthy controls is an important question for future studies. Nevertheless, the focus of the current study was on comparing different mood states in BD. Another important limitation was that it was not possible to analyze the effect of medication on memory. Most patients were on some medication, but the diversity of type and dosage prevented us from analyzing it. It is well-known that certain classes of medication interfere in memory function [e.g., benzodiazepines; (37)]. Future studies on autobiographical memory in BD with larger samples may benefit from categorizing patients according to medication status.

Understanding the impairment of autobiographical memory in patients diagnosed with schizophrenia helped in the development of clinical tools seeking to intervene and improve their ability to recall autobiographical memory (38–40). Increased knowledge about autobiographical memory in BD may lead to the development of clinical interventions with similar results to benefit patients with impaired ability to recall episodic and autobiographical events. In addition, current findings may improve the ability of clinicians to conduct psychiatric interviews and the diagnosis of BD, with special attention to how memory details are generated across different mood states of the condition. For example, our findings indicate depressed BD patients recall memories with fewer perceptual details and fragmented time integration, so this must be considered during clinical assessment, especially when prompting patients about the time sequence of events. By contrast, patients in mania generated memories with higher episodic detail, indicating this mood state may lead to more vivid re-experiencing, which may be used by clinicians when collecting information, teasing apart these details from the verbosity of the condition. Finally, the finding that words about BD provided greater episodic re-experiencing is also clinically relevant, suggesting that these can be used during the psychiatric anamnesis and also as memory prompts in future studies and therapeutic intervention (e.g., memory training).

## Data Availability Statement

The raw data supporting the conclusions of this article will be made available by the authors, without undue reservation.

## Ethics Statement

The studies involving human participants were reviewed and approved by Institute of Psychiatry, Federal University of Rio de Janeiro. The patients/participants provided their written informed consent to participate in this study.

## Author Contributions

RS and MT collected part of the data and wrote the manuscript. RL and RN collected part of the data. CS revised the manuscript. JL-F contributed to data analysis and revised the manuscript. AN revised the manuscript. EC supervised data collection, provided access to the patients, and revised the manuscript. DM designed the study, supervised data collection, conducted the analysis, and revised the manuscript. All authors contributed to the article and approved the submitted version.

## Conflict of Interest

The authors declare that the research was conducted in the absence of any commercial or financial relationships that could be construed as a potential conflict of interest.

## References

[1] Tulving E. Episodic memory: from mind to brain. Ann Rev Psychol. (2002) 53:1-25. 10.1146/annurev.psych.53.100901.135114 11752477

[2] Bluck S. Autobiographical memory: exploring its functions in everyday life. Memory. (2003) 11:113-23. 10.1080/741938206 12820825

[3] Dalgleish T Williams JMG Golden AMJ Perkins N Barrett LF Barnard PJ. Reduced specificity of autobiographical memory and depression: the role of executive control. J Exp Psychol Gen. (2007) 136:23-42. 10.1037/0096-3445.136.1.23 17324083 PMC2225543

[4] Young KD Bodurka J Drevets WC. Differential neural correlates of autobiographical memory recall in bipolar and unipolar depression. Bipolar Disord. (2016) 18:571-82. 10.1111/bdi.12441 27813234

[5] Riutort M Cuervo C Danion JM Peretti CS Salamé P. Reduced levels of specific autobiographical memories in schizophrenia. Psychiatry Res. (2003) 117:35-45. 10.1016/S0165-1781(02)00317-7 12581819

[6] Corcoran R Frith CD. Autobiographical memory and theory of mind: evidence of a relationship in schizophrenia. Psychol Med. (2003) 33:897-905. 10.1017/S0033291703007529 12877404

[7] McLeod H J Wood N Brewin CR. Autobiographical memory deficits in schizophrenia. Cogn Emot. (2006) 20:536-47. 10.1080/02699930500342472 26529221

[8] Serafini G Gonda X Canepa G Pompili M Rihmer Z Amore M. Extreme sensory processing patterns show a complex association with depression, and impulsivity, alexithymia, and hopelessness. J Affect Disord. (2017) 210:249-57. 10.1016/j.jad.2016.12.019 28064114

[9] American Psychiatric Association. Diagnostic and Statistical Manual of Mental Disorders (DSM-5^®^). Washington, DC: American Psychiatric Publishing (2013).

[10] Bosaipo NB Borges VF Juruena MF. Transtorno bipolar: uma revisão dos aspectos conceituais e clínicos. Medicina (Ribeirão Preto). (2017) 50(supl.1):72-84. 10.11606/issn.2176-7262.v50isupl1.p72-84

[11] Simon GE Hunkeler E Fireman B Lee JY Savarino J. Risk of suicide attempt and suicide death in patients treated for bipolar disorder. Bipolar Disord. (2007) 9:526-30. 10.1111/j.1399-5618.2007.00408.x 17680924

[12] Serafini G Canepa G Adavastro G Nebbia J Belvederi Murri M Erbuto D. The relationship between childhood maltreatment and non-suicidal self-injury: a systematic review. Front Psychiatry. (2017) 8:149. 10.3389/fpsyt.2017.00149 28970807 PMC5609590

[13] Bearden CE Glahn DC Monkul ES Barrett J Najt P Villarreal V. Patterns of memory impairment in bipolar disorder and unipolar major depression. Psychiatry Res. (2006) 142:139-50. 10.1016/j.psychres.2005.08.010 16631256

[14] Lewis PA Critchley HD. Mood-dependent memory. Trends Cogn Sci. (2003) 7:431-3. 10.1016/j.tics.2003.08.005 14550485

[15] King MJ MacDougall AG Ferris S Herdman KA Bielak T Smith JR. Impaired episodic memory for events encoded during mania in patients with bipolar disorder. Psychiatry Res. (2013) 205:213-9. 10.1016/j.psychres.2012.08.005 23237861

[16] American Psychiatric Association, DSM-5 Task Force. Diagnostic and Statistical Manual of Mental Disorders: DSM-5™. 5th ed. Arlington, VA: American Psychiatric Publishing, Inc. (2013).

[17] Hamilton M. A rating scale for depression. J Neurol Neurosurg Psychiatry. (1960) 23:56-62. 10.1136/jnnp.23.1.56 14399272 PMC495331

[18] Young RC Biggs JT Ziegler VE Meyer DA. A rating scale for mania: reliability, validity and sensitivity. Br J Psychiatry. (1978) 133:429-35. 10.1192/bjp.133.5.429 728692

[19] Spearing MK Post RM Leverich GS Brandt D Nolen W. Modification of the clinical global impressions (cgi) scale for use in bipolar illness (BP): the CGI-BP. Psychiatry Res. (1997) 73:159-71. 10.1016/S0165-1781(97)00123-6 9481807

[20] Levine B Svoboda E Hay JF Winocur G Moscovitch M. Aging and autobiographical memory: dissociating episodic from semantic retrieval. Psychol Aging. (2002) 17:677-89. 10.1037/0882-7974.17.4.677 12507363

[21] Park RJ Goodyer IM Teasdale JD. Categoric overgeneral autobiographical memory in adolescents with major depressive mood disorder. Psychol Med. (2002) 32:267-76. 10.1017/S0033291701005189 11866322

[22] Conway MA Pleydell-Pearce CW. The construction of autobiographical memories in the self-memory system. Psychol Rev. (2000) 107:261-88. 10.1037/0033-295X.107.2.261 10789197

[23] Williams JMG Scott J. Autobiographical memory in depression. Psychol Med. (1988) 18:689-95. 10.1017/S0033291700008370 3186869

[24] Burke M Mathews A. Autobiographical memory and clinical anxiety. Cogn Emot. (1992) 6:23-35. 10.1080/02699939208411056

[25] Wilhelm S McNally RJ Baer L Florin I. Autobiographical memory in obsessive-compulsive disorder. Br J Clin Psychol. (1997) 36:21-31. 10.1111/j.2044-8260.1997.tb01227.x 9051275

[26] Arntz A Meeren M Wessel I. No Evidence for overgeneral memories in bordeline personality disorder. Behav Res Ther. (2002) 40:1063-8. 10.1016/S0005-7967(01)00121-8 12296491

[27] Wessel I Meeren M Peeters F Arntz A Merckelbach H. Correlates of autobiographical memory specificity: the role of depression, anxiety and childhood trauma. Behav Res Ther. (2001) 39:409-21. 10.1016/S0005-7967(00)00011-5 11280340

[28] Harvey AG Bryant RA Dang ST. Autobiographical memory in acute stress disorder. J Consult Clin Psychol. (1998) 66:500-6. 10.1037/0022-006X.66.3.500 9642888

[29] McNally RJ Lasko NB Macklin ML Pitman RK. Autobiographical memory disturbance in combat-related posttraumatic stress disorder. Behav Res Ther. (1995) 33:629-30. 10.1016/0005-7967(95)00007-K 7654154

[30] McNally RJ Litz BT Prassas A Shin LM Weathers FW. Emotional priming of autobiographical memory in post-traumatic stress disorder. Cogn Emot. (1994) 8:351-67. 10.1080/02699939408408946

[31] Williams JMG Banhofer T Hermans D Raes F Watkins E Dagleish T. Autobiographical memory specificity and emotional disorder. Psychol Bull. (2007) 133:122-48. 10.1037/0033-2909.133.1.122 17201573 PMC2834574

[32] Wheeler MA Stuss DT Turving E. Toward a theory of episodic memory: the frontal lobes and autonoetic consciousness. Psychol Bull. (1997) 121:331-54. 10.1037/0033-2909.121.3.331 9136640

[33] Mansell W Lam D. A preliminary study of autobiographical memory in remitted bipolar and unipolar depression and the role of imagery in the specificity of memory. Memory. (2004) 12:437-46. 10.1080/09658210444000052 15487540

[34] Del Porto JA. Conceito e diagnóstico. Rev Bras Psiquiatr. (1999) 21:6-11. 10.1590/S1516-44461999000500003

[35] Palheta Neto FX. Anormalidades sensoriais: olfato e paladar. Arquivos Int. Otorrinolaringol. (2011) 15:350-8. 10.1590/S1809-48722011000300014

[36] Thones S Oberfeld D. Time perception in depression: a meta-analysis. J Affect Disord. (2015) 1:359-72. 10.1016/j.jad.2014.12.057 25665496

[37] Crowe SF Stranks EK. The residual medium and long-term cognitive effects of benzodiazepine use: an updated meta-analysis. Arch Clin Neuropsychol. (2018) 33:901–11. 10.1093/arclin/acx120 29244060

[38] Neshat-Doost HT Dalgleish T Yule W Kalantari M Ahmadi SJ Dyregrov A. Enhancing autobiographical memory specificity through cognitive training: an intervention for depression translated from basic science. Clin Psychol Sci. (2013) 1:84-92. 10.1177/2167702612454613

[39] Blairy S Neumann A Nutthals F Pierret L Collet D Philippot P. Improvements in autobiographical memory in schizophrenia patients after a cognitive intervention. Psychopathology. (2008) 41:388-96. 10.1159/000155217 18787361

[40] Heeren A Van Broeck N Philippot P. The effects of mindfulness on executive processes and autobiographical memory specificity. Behav Res Ther. (2009) 47:403-9. 10.1016/j.brat.2009.01.017 19232573

